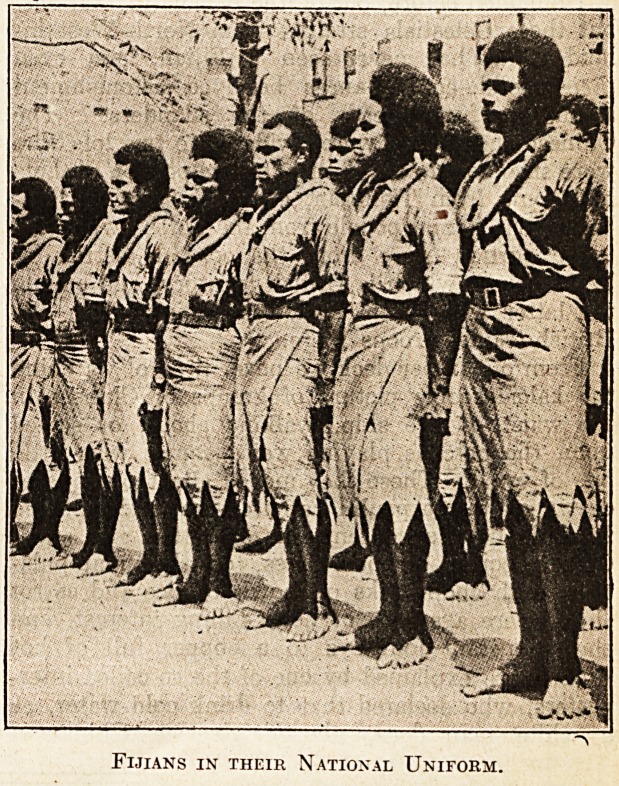# Many Races at the War

**Published:** 1918-10-26

**Authors:** 


### MANY RACES AT THE WAR.

### I.—With Native Labour Corps in France.

By A MEDICAL OFFICER.

Five years ago Macaulay's Maori gazing on the ruins of London would have seemed a vision just about as likely to happen as that of a Fiji Islander gazing pensively on the ruins of a town in France, a sight that I saw not so very long since in a much-raided area behind the lines. That remote murder in Serbia has indeed brought about an upheaval of the world! Fijians, Chinamen, Egyptians, Basutos, men of all races and of all creeds, have flocked to the standards of the Allies in the great struggle against darkness and oppression, and among one's interesting hospital experiences in France there vividly stands out the meeting with these people from far-off lands in the hospitals that have been set aside for their comfort and welfare.

Indian in Tropical Uniform.

It was my good fortune to come over from their own country with a contingent of Fijians, who were joined *en route* by a vast crowd of Chinese, all hurrying along the same path to the seat of war; while on arrival in France I was stationed at a big hospital where Egyptians, "Cape-boys," and West Indians were also treated, so that I was able to come in close touch with all these adventurers from the farthest corners of the earth, and some of my experiences as a medical officer among them I now set down.

Even the voyage over was brimful of new sensations. Imagine the first contingent of long-haired South Sea Islanders to go to the war! Such fine big men with smooth, light-brown skins, clear eyes, and proud carriage, these sons of the coral-reefs and born sailors had volunteered for special work on the ships that ceaselessly came from the four corners of the earth to the ports in France. Their duties were to unload the munitions, the foodstuffs, and the thousand-and-one articles required in modern warfare, and it was quickly found that at anything to do with a ship, from lifting off the hatches to working the steam winches, there was no one to compare with them. Early in the voyage their readiness and skill were put to the test, for on the big ocean liner that was carrying them across the Pacific there arose an alarm of fire, a serious matter when it was known that most of our cargo consisted of resin gum and New Zealand flax, and that it had become overheated! The Fijians were called upon, and, delighted to have something to do, they cleared out one of the holds, removed the source of danger, and re-stowed the cargo within a record time of six hours! I had my first casualty to deal with that afternoon, fortunately not a very serious one, a crushed toe. Such accidents were at first common, as the full uniform of the Fijians was a khaki shirt, a vandyked kilt of the same material, no

Fijians in their National Uniform.

boots or stockings, and of course no hat. But it was found that in the rough streets of French towns boots and stockings were a necessity, and in the colder weather breeches were issued in exchange for the national sulu or kilt.

At Honolulu the Fijians had a great reception, as the Americans had just thrown in their lot with the Allies, and we were the first British troops passing through since that event. The Honolulu native band played us through the town, speeches were made, and a luncheon was provided out at the famous Waikiki beach hotel, near the Zoological Gardens; where the Fijians saw and had rides upon their first elephant, an awe-inspiring animal, even though it was only a baby one!

A day or two after leaving Honolulu one of the

**Many Races at the War—(*continued*).**

Fijians, a man rejoicing in the name of Eparama or Abraham, developed pneumonia; and by the time we reached Vancouver he was very ill indeed. I had him taken into the big general hospital there, and as it happened that we were held up by traffic difficulties for eleven days, we were able to take him on with us, convalescent, when we left.

This hospital was an eye-opener to the Fijians (many of whom used to come up to sit with their friend), as it was the first big building of its kind they had ever seen. It is indeed a splendidly equipped hospital, complete with the latest devices of every imaginable kind, and now has a large military annexe built on to it.

At Vancouver we first fell in with the Chinese, also *en route* to the great struggle in Europe, and the two races stared at each other in frank amazement. The Fijians had seen scattered handfuls of Chinamen before, out on the banana plantations, but these Celestials straight from North-West inland China had never seen a Fijian, and even *their* stolid countenances betrayed astonishment at the big fuzzy heads of the islanders. The Chinese themselves were divided into two lots who were strangers to each other, as one portion of them had come from Tien-tsin, at which port had embarked the inland people from the north; while the other portion were from the coastal district of Wei-hai-wei, which is British territory, and among the latter were even some ex-soldiers from the British-trained troops there.

From our upper deck we never tired of watching the kaleidoscopic picture of groups of Chinese in the waist of the ship, walking about, or seated upon the hatches playing weird games with little bits of wood. These they moved about on squares drawn on the tarpaulin hatch-covers, and gambled to their heart's content for cigarettes and small Chinese coins. At meal-times, too, their manipulations with chop-sticks on bowls of rice and carrot slices were a source of never-failing interest, and their constant journeys to a copper full of hot water were explained by one of the so-called interpreters, who declared that to drink cold water, as a white man does, always gives a Chinaman "muchee belly pain" and even dysentery! We certainly had a case of dysentery between Quebec and Halifax, and had to put the man, one of the Wei-hai-wei lot, ashore at the hospital there; but I hardly think the interpreter's view of the cause was quite sound all the same!

It was greatly due to the care of the medical officer who was in charge of the Chinese that the dysentery was checked with this one case, as we had between two and three thousand of them on this troopship (imagine the scene if we had been torpedoed!), and naturally the accommodation was somewhat scanty and cleansing facilities limited. Each Chinaman was provided with two blue quilted suits, which he wore one on the top of the other when the weather turned cold; and on such occasions he also turned out with his funny little round cap with the two ear-pieces of grey rabbit's fur let down and tied under his chin. The blue suits were all marked with consecutive red-embroidered numbers, running into many tens of thousands, on the epaulettes; and the man kept to that number all through his service in France. He was not, however, beyond occasionally trying to wear another man's uniform and draw his pay as well as his own, but this was checked by the finger-print system, every man in the vast Chinese Labour Corps being thus recorded.

On the whole, the Chinese on that voyage were a very well-behaved lot, largely due to their having for their officers Englishmen who had lived amongst them for many years in China, and thoroughly understood their language and customs. Any legitimate attempts to amuse themselves were much encouraged, and a comic actor or two who happened to be among them were afforded every opportunity to display their art, as a Chinaman is in many ways like a child and always amenable if kept amused. Another man we used to enjoy watching was a musician who drew weird noises from a Chinese one-stringed fiddle, accompanying the music with a shuffling dance. His droll and much-wrinkled face showed the born comedian, and easily explained the roars of laughter that always greeted his appearance on the scene.

(*To be continued.*)

## Figures and Tables

**Figure f1:**
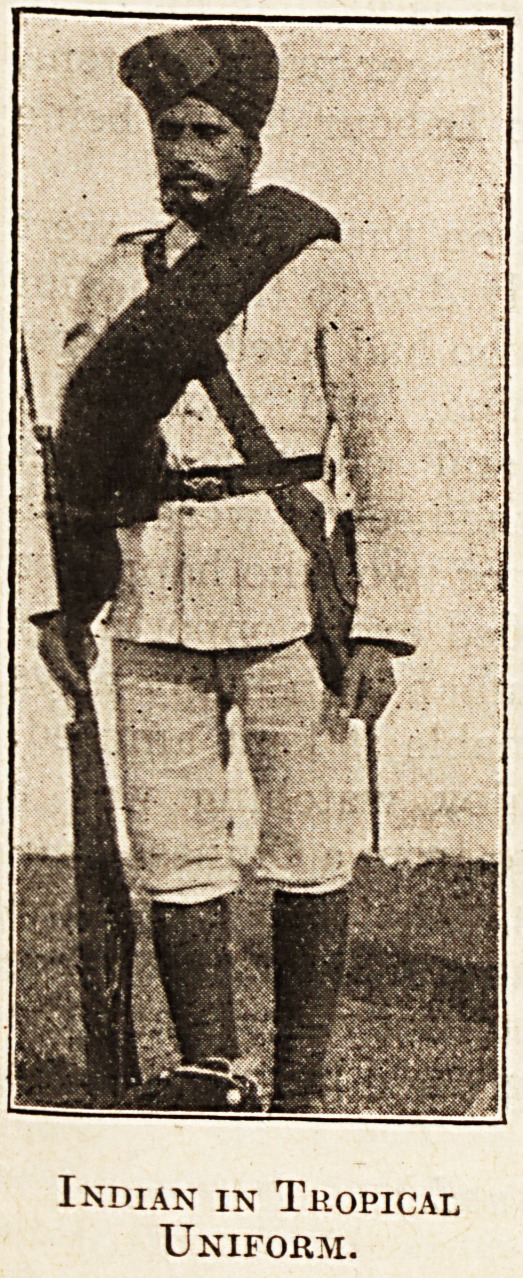


**Figure f2:**